# Supplementary material for: Identification and functional analysis of non-coding regulatory small RNA FenSr3 in Bacillus amyloliquefaciens LPB-18
Source: PeerJ. 2023 May 15;11:e15236. doi: 10.7717/peerj.15236 (PMC10194069; doi:10.7717/peerj.15236)
Supplement: Supplemental Information 4 [file peerj-11-15236-s004.zip › KO/CK-vs-T1_map/map04726.html]

KEGG PATHWAY: Serotonergic synapse - Reference pathway


|  |  |
| --- | --- |
| **Serotonergic synapse - Reference pathway** |  |

[
Pathway menu
| Organism menu
| Pathway entry
| Show description
| User data mapping
]

Serotonin (5-Hydroxytryptamine, 5-HT) is a monoamine neurotransmitter that plays important roles in physiological functions such as learning and memory, emotion, sleep, pain, motor function and endocrine secretion, as well as in pathological states including abnormal mood and cognition. Once released from presynaptic axonal terminals, 5-HT binds to receptors, which have been divided into 7 subfamilies on the basis of conserved structures and signaling mechanisms. These families include the ionotropic 5-HT3 receptors and G-protein-coupled 5-HT receptors, the 5-HT1 (Gi /Go -coupled), 5-HT2(Gq-coupled), 5-HT4/6/7 (Gs-coupled) and 5-HT5 receptors. Presynaptically localized 5-HT1B receptors are thought to be the autoreceptors that suppress excess 5-HT release. 5-HT's actions are terminated by transporter- mediated reuptake into neurons, leading to catabolism by monoamine oxidase.

|  |  |  |
| --- | --- | --- |
| Reference pathway | 184% 150% 122% 100% 82% 67% 55% | 图片下载 |
